# Impact of TNF inhibitor medication on working ability in axial spondyloarthritis: an observational national registry-based cohort study

**DOI:** 10.1093/rap/rkad050

**Published:** 2023-05-25

**Authors:** Anna-Mari Hokkanen, Kalle Aaltonen, Heikki Relas, Jarno Rutanen, Aulikki Kononoff, Kirsi Taimen, Markku Kauppi, Kari Puolakka, Nina Trokovic, Dan Nordström

**Affiliations:** ROB-FIN Register, Division of Rheumatology, Helsinki University Hospital, Helsinki, Finland; Division of Rheumatology, Päijät-Häme Central Hospital, Lahti, Finland; Pharmaceuticals Pricing Board, Ministry of Social Affairs and Health, Helsinki, Finland; Division of Rheumatology, Helsinki University Hospital, Helsinki University, Helsinki, Finland; Unit of Health Sciences, Faculty of Social Sciences, Tampere University, Tampere, Finland; Centre for Rheumatic Diseases, Tampere University Hospital, Tampere, Finland; Division of Medicine, University Hospital, Kuopio, Finland; Division of Medicine, Turku University Hospital, Turku, Finland; Division of Rheumatology, Päijät-Häme Central Hospital, Lahti, Finland; Division of Rheumatology, Helsinki University, Helsinki, Finland; Helsinki Rheumatic Diseases and Inflammation Research Group, University of Helsinki, Helsinki, Finland; ROB-FIN Register, Division of Rheumatology, Helsinki University Hospital, Helsinki, Finland; ROB-FIN Register, Division of Rheumatology, Helsinki University Hospital, Helsinki, Finland

**Keywords:** work disability, TNF inhibitor, axial SpA, AS, non-radiographic axial SpA, register study

## Abstract

**Objective:**

The aim was to investigate the effect of TNF inhibitor (TNFi) initiation on working ability and health-care resource utilization among axial SpA patients in a real-life setting.

**Methods:**

Patients with a clinical diagnosis of non-radiographic (nr-axSpA) or radiographic axial SpA initiating their first TNFi were identified from the National Register for Antirheumatic and Biologic Treatment in Finland. Sickness absences, including sick leave and disability pension, in- and outpatient days and rehabilitation rates, 1 year before and after initiating the medication were retrieved from national registries. Factors affecting result variables were studied using multivariate regression analysis.

**Results:**

Overall, 787 patients were identified. Rates of work disability days per year were 55.6 the year before treatment onset and 55.2 the year after, with significant differences between patient subgroups. The rate of sick leave decreased after starting TNFi treatment. However, the rate of disability pension continued to rise. Patients with a diagnosis of nr-axSpA experienced a decrease in overall work disability and, especially, fewer sick leaves. No sex differences were detected.

**Conclusion:**

TNFi interrupts the increase in work disabled days evident during the year before its initiation. However, the overall work disability remains high. Treating patients earlier in the nr-axSpA phase, regardless of sex, appears important in maintaining the ability to work.

Key messages

- Rise of absenteeism subsided with the initiation of TNFi.
- Beneficial effect on work absenteeism was more pronounced with the diagnosis of non-radiographic axial SpA regardless of sex.
- Long-term work disability increased despite TNFi initiation.

## Introduction

AS and non-radiographic axial SpA (nr-axSpA) are inflammatory rheumatic diseases. These conditions initiate typically before the age of 40 years, and AS patients have been shown to have increased working disability and health-care resource use in comparison to the general population [1–5]. The inflammatory activity varies over time, but delayed diagnosis and insufficient treatment can lead to considerable disability [6–9], as also highlighted by van Hoeven *et al.* [5]. Delayed diagnosis was connected to unemployment in a recent study in the USA [10]. The main cause of disability is spinal stiffness and pain. In addition, peripheral arthritis and uveitis often occur, sometimes also with other extra-articular manifestations, such as IBD. Co-morbidities, such as fatigue and depression, are also present. Loss of effective working years results in indirect costs to society [4, 7, 8, 11–13].

nr-axSpA was recognized as an entity in 2009 [14], and since 2013 the diagnosis has entitled patients in Finland to biological drug reimbursement, if traditional anti-rheumatic drugs are insufficient to control the disease. In Finland, the incidence of AS has been reported to be 7.3 per 100 000 and unspecified SpA 18 per 100 000 [15]. The incidence rate for all SpA has risen from 12 to 18 per 100 000 between 2000 and 2014 [16, 17].

Work ability is an important goal when treating patients of working age. The expansion of medication alternatives and progress in treatment strategies are expected to improve work capability and employment. TNF inhibitors (TNFi) have been proved to be clinically beneficial, safe and cost effective in both AS and nr-axSpA [18, 19]. Evidence from clinical trials suggests that TNFi are able to prevent work disability in patients with AS [20], but these findings might not be generalizable to routine clinical practice owing to the stringent inclusion criteria for clinical trials [21]. Results from the British Society for Rheumatology biologics register and meta-analysis show significant improvement in work productivity, although absenteeism was seen to remain high [22].

The aim of this study was to evaluate the effect of starting a TNFi on absenteeism and the use of health-care resources in a national cohort of axSpA patients including both nr-axSpA and AS patients.

## Methods

### Patients

Patients were identified from the National Register for Antirheumatic and Biologic Treatment in Finland (ROB-FIN), which is a longitudinal cohort study with data dating back to 1999. ROB-FIN was established initially to monitor the effectiveness and safety of biologic DMARDs (bDMARDs) in rheumatic diseases. Inclusion for this study was limited to TNFi treatments started as the patient's first bDMARD treatment between 2007 and 2015.

According to the Finnish treatment guidelines and the restricted reimbursement confirmed by the Finnish Pharmaceuticals Pricing Board, patients with AS and nr-axSpA must be refractory to at least one conventional synthetic DMARD (csDMARD) before they are entitled to reimbursement for bDMARDs.

We included patients with a clinical diagnosis of AS or nr-axSpA (ICD10 diagnosis codes M45 and M46). At the start of TNFi therapy, baseline characteristics were reported by the treating physicians using a standardized protocol. The data were collected with paper forms or by applying the GoTreatIt computer software platform. Missing baseline data were imputed by multiple imputation with predictive mean matching and 10 imputed data sets.

### Outcomes and follow-up

The study was conducted as an observational cohort study. The outcomes of interest in this study comprised days absent from work owing to sick leave, disability pension or rehabilitation and the number of inpatient days and outpatient visits to specialized health-care facilities.

The Finnish social insurance system provides financial support for individuals of working age in the event of illness or injury. We defined sick leave as days with sickness allowance of any degree paid by the Social Insurance Institution of Finland (SII). All sick leaves with a duration of ≥10 days are registered continuously and prospectively by the SII. Sick leaves shorter than this are not registered, because the employer provides the pay. Sick leave is paid for ≤300 days. After this, the patient can apply for a fixed-term rehabilitation subsidy, which is granted for the time it is needed for medical and/or vocational rehabilitation to restore the ability to work. If the rehabilitation measures are not able to restore working ability, the patient applies for a disability pension. In our study, we viewed sickness absences as temporary leaves (sick leave and rehabilitation subsidy) and as permanent incapacity to work, i.e. disability pension.

Information on sick leaves were retrieved from the SII, whereas the data on pensions were gathered from SII and Finnish Centre for Pensions. The information on the outpatient and inpatient visits was gathered from Care Registers for Social Welfare and Health Care. The data on deaths and emigrations were retrieved from Statistics Finland.

The baseline visit took place when TNFi treatment was initiated. The follow-up time was 1 year before and 1 year after the start of a TNFi. However, follow-up was terminated in the event of death or emigration. When analysing sick leaves and disability pensions, the patients were also censored when they became pensioners (excluding disability pension) or turned 63 years old, which is the common retirement age in Finland.

A favourable ethical board statement was granted by the Helsinki and Uusimaa Hospital District coordinating ethical committee (73/13/03/00/2014), and permission for the study was obtained from the National Institute for Health and Welfare (THL/6752/14.06.00/2020). Written informed patient consent were acquired from patients who had been included in ROB-FIN before the introduction of the electronic patient monitoring systems covered by the permission above.

### Statistical methods

Baseline characteristics at initiation of TNFi treatment are presented as the mean along with standard deviations or percentages. The outcomes are reported as events per patient-year, with the 95% CI calculated via non-parametric bootstrapping. The missing values among patient characteristics were imputed using a multiple imputation method. The annual change in the rate of the outcomes before and after the initiation of TNFi was modelled with linear regression, with an additional error term for repeated measures. The explanatory variables included age, sex, HLAB27, CRP, ASDAS-CRP, TNFi agent, concomitant use of csDMARDs and the year of treatment onset. The year of treatment onset was divided into three periods of equal length (2007–2009, 2010–2012 and 2013–2015) to examine whether the availability of new TNFi drugs (certolizumab pegol and golimumab) and the expansion of medication use would have an effect on work outcome. Data management and statistical analyses were carried out using R software v.3.6.2 (R Foundation for Statistical Computing, Vienna, Austria).

## Results

### Patients and follow-up

Overall, 787 patients were included in the study, of whom 684 and 103 were diagnosed with AS and nr-axSpa, respectively. A full year worth of follow-up was available for 783 patients, whereas 4 were censored during the follow-up owing to death or emigration. Of the overall population, 734 were of working age the year before initiation of TNFi, and among this subgroup an additional 27 patients were censored owing to retirement, death or emigration.

Baseline characteristics are described in Table 1. The mean age was 40 years, and 56% of the patients were men. The mean DAS according to ASDAS was 3.0 and BASDAI 44. Patient global assessment was 47 and investigator global assessment 35. Mean baseline ESR was 17 mm/h, and CRP was 13 mg/l. The AS patients were predominantly male (59%), whereas nr-axSpa patients were mostly female (64%). The time from diagnosis to starting treatment was 4.2 (s.d. 5.5) years for nr-axSpA and 7.1 (s.d. 7.8) years for AS.

**Table 1.** SpA patient characteristics at baseline

Variable	Mean (s.d. or percentage)		
	All patients (*n* = 787)	AS (*n* = 684)	Nr-axSpA (*n* = 103)
Age, years	40 (11)	40 (11)	38 (11)
Sex	442 (56% male)	405 (59% male)	37 (36% male)
Weight, kg	79 (16)	79 (15)	80 (18)
Height, cm	172 (10)	173 (9.4)	171 (9.1)
Patient global assessment	47 (26)	47 (25)	48 (25)
Investigator global assessment	35 (22)	35 (20)	30 (19)
HAQ	0.70 (0.5)	0.71 (0.49)	0.68 (0.51)
Pain	52 (25)	52 (26)	51 (23)
ASDAS	3.0 (1.0)	3.0 (1.0)	2.8 (1.1)
BASDAI	44 (20)	44 (21)	44 (19)
ESR, mm/h	17 (18)	17 (17)	14 (16)
CRP, mg/l	13 (17)	13 (16)	11 (15)
Time from diagnosis, years	6.7 (7.6)	7.1 (7.8)	4.2 (5.5)

At the initiation of TNFi medication, 73% of the patients received concomitant csDMARD treatment, while the remaining 27% of the patients received biologic monotherapy. MTX was used by 46% of the patients and SSZ by 41% . Oral low-dose glucocorticoids were used by 27% of the patients. Adalimumab was the most frequently used TNFi, by 35% of the patients, followed by etanercept (30%), infliximab (22%) golimumab (10%) and certolizumab pegol (1.9%) (Table 2).

**Table 2.** Medication when first TNF inhibitor initiated

Medication	*n* (%)
TNF inhibitor	
Adalimumab	277 (35)
Etanercept	238 (30)
Infliximab	175 (22)
Golimumab	82 (10)
Certolizumab pegol	15 (1.9)
Concomitant medication	
Any conventional DMARD	577 (73)
MTX	357 (46)
SSZ	322 (41)
Glucocorticoids	161 (27)
Other conventional DMARD	78 (9.8)

### Outcomes

**Figure 1:**
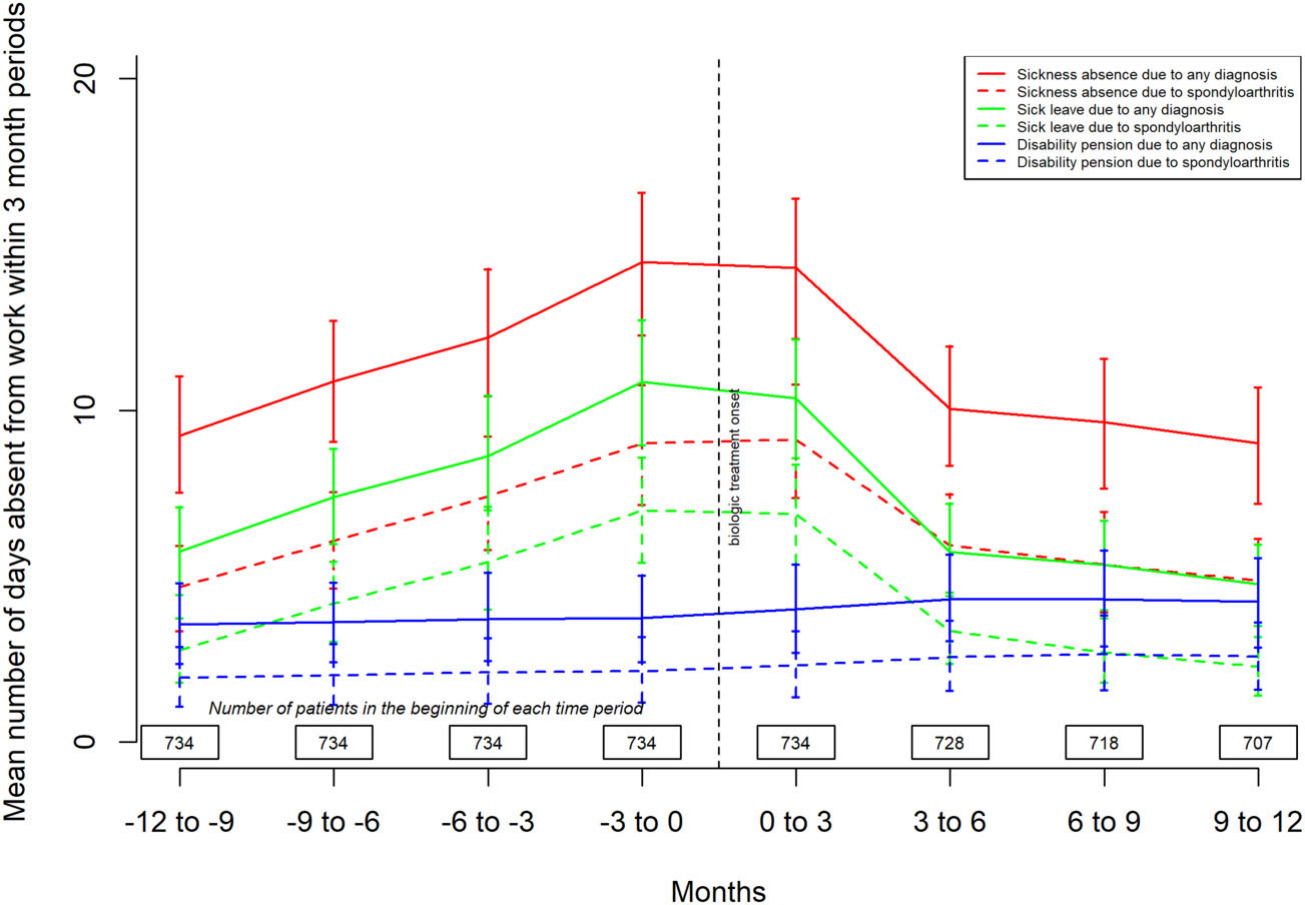
Number of days absent from work as 90-day rates. Sick leave, disability pension and pooled work disability 1 year before and after TNF inhibitor treatment onset (i.e. baseline) among the patients of working age

The annual number of sick disabled days decreased slightly in the overall population: from 47.3 (95% CI 40.9, 53.8) days during the year before TNFi initiation to 43.6 (95% CI 36.5, 50.8) days the year after (Table 3). The 90-day rate of work absence days increased during the first year of follow-up until TNFi initiation and decreased thereafter (Fig. 1).

**Table 3.** Crude annual incidence rates of outcome variables by diagnosis

	Days during 1 year pre-TNFi [95% CI]	Days during 1 year post-TNFi [95% CI]	Days during 1 year pre-TNFi [95% CI]	Days during 1 year post-TNFi [95% CI]	Days during 1 year pre-TNFi [95% CI]	Days during 1 year post-TNFi [95% CI]
			
Incidence rate	AS (*n* = 640)		nr-AxSpA (*n* = 94)		All (*n* = 734)	
Rate of work disabled days	48.6 [41.2, 56.5]	46.6 [39.3, 54.1]	38.2 [23.5, 55.6]	22.83 [12.1, 36.7]	47.3 [40.9, 53.8]	43.6 [36.5, 50.8]
Rate of sick leave days, all causes	32.1 [27.0, 37.7]	28.1 [23.5, 33.3]	38.2 [23.5, 55.6]	16.1 [8.9, 24.8]	32.90 [27.9, 38.1]	26.5 [22.4, 31.1]
Rate of sick leave days, SpA	19.6 [15.0, 24.3]	16.61 [12.9, 21.2]	18.8 [7.8, 32.9]	7.4 [3.0, 13.2]	19.5 [15.5, 23.6]	10.3 [6.1, 14.7]
Rate of disability pension days, all causes	16.9 [11.5, 23.3]	18.7 [12.8, 24.5]	0 [0.0, 0]	6.7 [0.0, 17.6]	14.8 [9.7, 19.7]	17.1 [11.4, 22.7]
Rate of disability pension days, SpA	9.5 [5.5, 14.2]	11.35 [7.0, 15.9]	0 [0.0, 0]	3.1 [0.0, 9.9]	8.3 [4.86, 11.9]	10.3 [6.1, 14.7]

	**AS (*n* = 684)**		**nr-AxSpA (*n* = 103)**		**All (*n* = 787)**	

Rate of outpatient visits (visits per year)	6.4 [5.9, 6.8]	7.2 [7.00, 7.5]	7.6 [6.54, 8.6]	6.4 [6.0, 8.1]	6.5 [5.1, 6.9]	7.4 [6.9, 7.8]
Rate of inpatient days (days per year)	1.0 [0.8, 1.3]	1.1 [0.8, 1.3]	1.2 [0.6, 1.9]	1.6 [1.0, 2.3]	1.6 [1.3, 2]	1.5 [1.3, 1.9]

nr-AxSpA: non-radiographic axial SpA.

The rate of days on sick leave decreased after TNFi initiation within the subgroup of patients of working age (Fig. 1). The incidence of sick leaves during the first and second time periods in this subgroup was 32.90 (95% CI 27.9, 38.1) and 26.5 (95% CI 22.4, 31.1), respectively (Table 3). Meanwhile an increasing trend for days absent from work owing to disability pensions was observed in the overall population: from 14.8 (95% CI 9.7, 19.7) to 17.1 (95% CI 11.4, 22.7) days. The same trend was seen both in AS and nr-axSpA patients.

Absenteeism reduced to a larger extent among the nr-axSpA patients, from 38.2 (95% CI 23.5, 55.6) days per patient year the year before to 22.83 (95% CI 12.1, 36.7) days per patient year the year after. Among AS patients, the values changed from 48.6 (95% CI 41.2, 56.5) to 46.6 (95% CI 39.3, 54.1) days per patient year.

**Figure 2:**
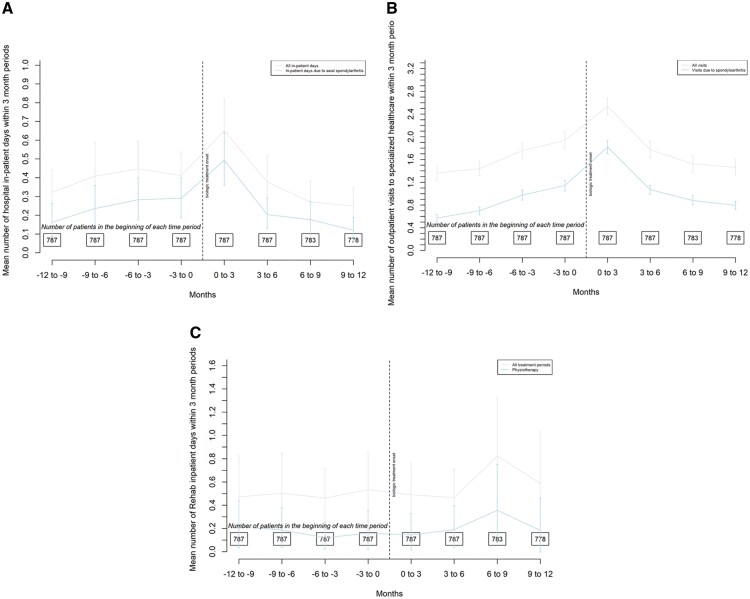
Ninety-day rates 1 year before and after TNF inhibitor treatment onset. (**A**) Hospital in-patient days. (**B**) Health-care visits in specialized health care. (**C**) Rehabilitation days.

No statistically significant changes were observed in health-care resource utilization between the two periods (Fig. 2). The rates of inpatient days and outpatient visits were similar before and after the initiation of TNFi therapy.

Of the 48 patients who were absent from work for the whole year before TNFi treatment began, only 7 (15%) returned to work during the 1-year follow-up. Additionally, 17 patients who were working at least part time during the year before treatment initiation were absent for the entire subsequent year after treatment initiation.

### Regression analyses

**Figure 3:**
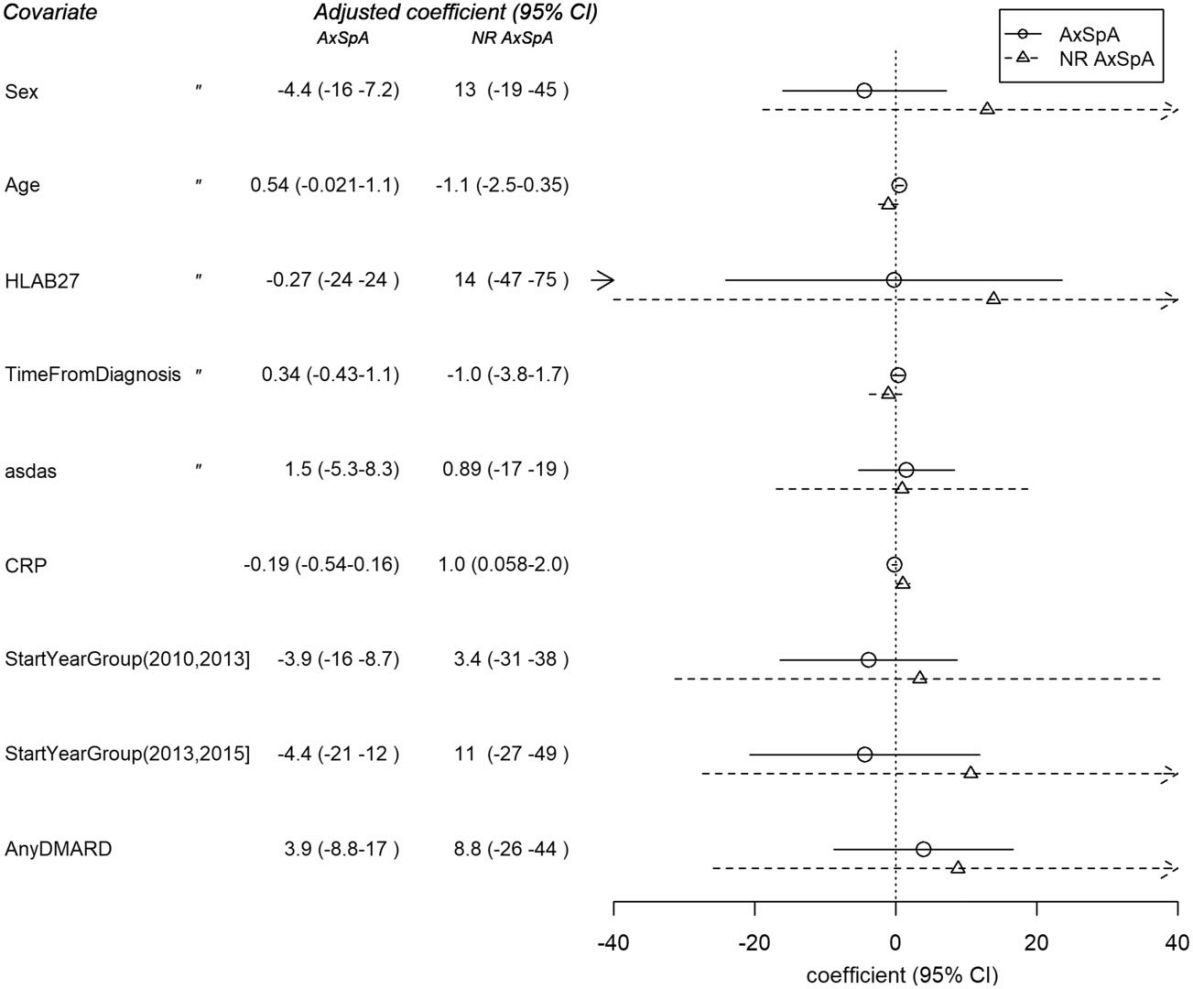
Correlation coefficients between explanatory variables and the change in annual rates of work disabled days. AxSpA: Ankylosing spondylitis; NR AxSpa: Non-radiographic spondyloarthritis

Based on the linear regression analysis, disease activity at baseline (ASDAS-CRP) did not demonstrate a statistically significant association with the annual change in absenteeism, nor did HLAB27, HAQ, smoking or BMI. Even age did not seem to change the outcome; CRP was associated with a modest increase in the number of days absent from work among nr-axSpA patients, but not in AS patients (1.06, 95% CI 0.058, 2.0 *vs* −0.19, 95% CI 0.16, 3.2; Fig. 3). However, the small values do not appear clinically significant. The choice of TNFi agent was not a statistically significant predictor of improvement in absenteeism, aside from certolizumab pegol reaching statistical significance in comparison to adalimumab among axSpA patients (64, 95% CI 18, 110), but this was probably attributable to bias caused by the small number of patients in the certolizumab pegol group (15 patients). However, the decrease in absenteeism in the nr-axSpA population was significantly more pronounced than in the AS population (*P* = 0.039) and also when age, sex, HLAB27 status, time from diagnosis and CRP were standardized (*P* = 0.056).

## Discussion

Our results showed that the rate of sick leaves decreased notably after the initiation of TNFi treatment in the nr-axSpA group. Meanwhile the rate of disability pension days continued to rise slightly throughout both follow-up years. The number of sick leave days remained high in the AS group. A lack of improvement in overall work absence was shown previously by a British observational study [22]. However, in this study the nr-axSpA patients benefitted especially from the TNFi treatment.

The need for inpatient care was low, although there was a slight increase in inpatient days around the beginning of drug treatment. The non-significant increase in outpatient visits during the follow-up year after initiating TNFi treatment compared with the year before TNFi is probably because the baseline visit was included in the former. Defining the follow-up period in this way was intentional because prescribing the new treatment warrants a visit to a rheumatologist.

ASAS/EULAR guidelines recommend TNFi as a second-line treatment, if disease activity remains high despite NSAID therapy [23]. In Finland, SII requires a treatment attempt with cDMARDs before reimbursement for TNFis is granted. Patients treated with TNFi are the subgroup of most severe cases, because patients with a milder disease course are treated primarily with NSAIDs or cDMARDs. One of the reasons for these Finnish treatment guidelines is that Finnish patients using co-medication with cDMARDs and biologics show added beneficial effect [24, 25]. Moreover, recent international register data have replicated these findings [26]. These studies shed light on the disparity of results on this subject across publications from different national registries over the past decade. They also suggest that, in contrast to current international treatment guidelines, csDMARD co-therapy should not necessarily be disregarded in axSpA. However, in the present study, co-medication with csDMARD did not show added benefits in terms of working ability.

The TNFi are shown to be effective in both AS and nr-axSpA, when objective signs of inflammation are present [27]. In nr-axSpA with no objective signs of inflammation, the efficacy has been shown to be lower [28, 29]. The association between CRP levels and an increase in the days absent from work is in contrast to our expectations of patients with higher disease activity gaining more benefit from TNFis. The finding was, however, only borderline significant in the nr-axSpA group and absent in the AS group, which is why we refrain from drawing any definite conclusion on this topic.

We discovered that despite initiation of TNFi, the amount of disability pensions rises steadily. The results emphasize the importance of managing the disease in a multidisciplinary fashion, managing pain and taking work ability and rehabilitation into consideration early in the disease course.

There is little evidence of non-pharmacological interventions improving employment among patients with SpA [30]. In a Swedish study, the sick leave and disability pension rates remained higher in SpA patients than in the general population despite pharmacological interventions [31]. TNFi therapy combined with physiotheraphy could offer a synergistic effect [32]. More research is needed on the possibilities of enhancing work rehabilitation of patients with SpA [33–35].

SpA also causes decreased presenteeism, work productivity loss and inability to succeed in daily unpaid activities, as seen in research using patient surveys [4, 13, 36, 37]. Unfortunately, we did not have the tools to examine these aspects in our study.

There have been studies considering work-related issues related to sex, evaluating differences between males and females in the treatment response to TNFi [10]. Therefore, we included sex as a factor in the regression model. However, we did not detect a significant difference between males and females in the decrease of work disability in either the AS or the nr-axSpA population (Fig. 3).

Previous studies have observed correlations between disease activity and health-care resource use or work absence in rheumatic diseases, which have, in turn, been used as inputs for modelling cost-effectiveness of TNFis. Whether the treatment response achieved with TNFis manifests as savings in health-care costs and productivity losses can be analysed by examining the incidence of in- and outpatient visits and work absence days before and after the treatment initiation [38, 39]. Unlike the typical assumption in cost-effectiveness models, improved well-being of an individual might not lead to improved employment for all individuals if they are still eligible to enter or remain on disability pension. Notably, our results showed that only a fraction of patients who were on disability leave for the entire year before TNFi onset returned to work during the subsequent year. SpA patients are likely to experience other health-related concerns, such as fatigue, sleep and emotional problems [40]. These symptoms are not alleviated by TNFi therapy as well as inflammation-mediated pain and stiffness [41].

Our results showed an increasing trend in the incidence of work disability during the year before TNFi initiation. This is probably attributable to the worsening of symptoms, which eventually qualify the patient for reimbursement criteria for TNFis. Together with the selection criteria for the study and the temporal variability in symptom severity, this means that at the baseline of their first TNFi, the patients might be at the peak of their symptoms. The impact of the regression-to-the-mean phenomenon cannot be ruled out when interpreting the decreasing incidence of outcomes during the subsequent year.

Given that SpA has a chronic and progressive disease course, the short study period of 1 year after treatment initiation does not fully describe the effect of TNFi medication upon the individual’s health over the years [38]. It is reasonable to compare the years before and after the initiation of the TNFi treatment. In a Finnish study from 1987, the disability rate at 8 years was 15% since the diagnosis of AS. The major cause for limited working capacity was disease severity [42]. In another study from the 1980s, in which the natural disease course of AS was investigated, disability generally developed within first 10 years of disease [43]. After 25 years of AS, 48% of the patients were working in their original occupations and 30% were unable to work at all. Seventeen per cent of the patients were forced to change their occupation [44].

An advantage in this study is that missingness does not occur in endpoint data, because all sick leaves >10 days, disability pensions and hospital stays are collected from official records. However, patients who are waiting for a disability pension decision after 300 days of sick leave have a status of unemployment and are not recorded in the registries. Also, short (<10 working days) sick leaves are not recorded and thus were not included in the study.

As with many register data, there was extensive missingness in patient characteristics. Should some of the data be missing not at random, our attempts to impute it with predictive mean matching would potentially introduce some bias into the results.

In conclusion, TNFi treatment seemed to interrupt the increase in absenteeism evident during the year before their initiation among patients with AS or nr-axSpA. Given that the effect was more pronounced on the sick leaves alone, it was worth noting that the rates of days on disability pension continued to increase regardless of TNFi therapy. Patients with nr-axSpA diagnosis, regardless of sex, seem to benefit more from TNFi therapy in terms of avoiding work disability, which indicates a better effect earlier in the disease course. The results highlight the importance of managing the disease in a multidisciplinary fashion, managing pain and taking the work outcome and rehabilitation into consideration early in the disease course. This also justifies introducing the diagnosis of nr-axSpA over the more chronic AS diagnosis when trying to avoid work disability.

## Data Availability

The data underlying this article cannot be shared publicly owing to the need to protect the privacy of individuals who participated in the study. However, the code with which the data have been processed is available upon reasonable request to the corresponding author.
